# Perspective-taking is two-sided: Misunderstandings between people with Asperger’s syndrome and their family members

**DOI:** 10.1177/1362361317708287

**Published:** 2017-07-07

**Authors:** Brett Heasman, Alex Gillespie

**Affiliations:** The London School of Economics and Political Science, UK

**Keywords:** Asperger’s syndrome, family relationships, methodology, misunderstanding, mixed methods, perspective-taking

## Abstract

Misunderstandings are social in nature, always having two sides. Yet the misunderstandings experienced by people with Asperger’s syndrome are usually studied in terms of the individual with a diagnosis, with less emphasis on social relations. We use a two-sided methodology to map out misunderstandings within 22 dyads (n = 44) consisting of people with Asperger’s syndrome and their family members. Both sides of the relationship were asked about 12 topics in terms of one’s rating of Self, one’s rating of Other and one’s predicted rating by Other. The findings show that people with Asperger’s are able to predict lower scores from family members, despite disagreeing with their view, and that family members often over-estimate the extent to which their relatives with Asperger’s syndrome are egocentrically anchored in their own perspective. The research demonstrates that a two-sided methodology is viable, and it uses it to identify how representations of Asperger’s syndrome can both support and hinder social understanding within relationships affected by Asperger’s.

## Introduction

Although people diagnosed with Asperger’s syndrome (AS) report difficulty in understanding what other people are thinking (Hochhauser et al., 2015; Locke et al., 2010; Muller et al., 2008), research has also shown that in social relations this phenomenon is two-sided, because close friends and family also have difficulty in understanding people with Asperger’s syndrome (PwAS; Brewer et al., 2016; Froese et al., 2013; Kremer-Sadlik, 2004). Misunderstandings (when one party attributes an incorrect belief to another party) are therefore experienced by both people with AS and their relations, and as such, it is important to develop methods for investigating the two-sided nature of these misunderstandings. While ethnography has been productively used to explore the two-sided nature of these relationships (Maynard, 2005; Ochs, 2010; Solomon, 2010), there is currently a lack of methods used in research on AS that systematically compares the perspectives of each side within real social relationships.

We report research based on the Interpersonal Perception Method (IPM), a two-sided methodology for identifying how members of a given social relation understand or misunderstand each other. Through a rating exercise and open-ended discussion, the IPM methodology systematically compares direct perspectives (one’s view of Self and one’s view of Other) and meta-perspectives (how one thinks one is seen by Other), and provides a basis for interpreting the origins of misunderstanding. We used the IPM methodology to examine relationships involving participants from a charity supporting PwAS and their family members (FMs). The IPM was used to examine: what misunderstandings occur in PwAS–FM relationships? And what reasons do participants give for such misunderstandings?

### Misunderstandings in relationships with people with Asperger’s

Misunderstandings in PwAS–FM relationships may be two-sided (i.e. evident for both people with AS and their relatives) for cognitive, social and cultural reasons. Cognitive reasons for misunderstanding are well documented, highlighting how the individuals with AS may struggle to make themselves appropriately ‘readable’ to others because of limitations in theory of mind (Bowler, 1992; Spek et al., 2010), executive control (Ozonoff et al., 1991; Pellicano et al., 2006), emotion perception and regulation (Montgomery et al., 2013; Samson et al., 2012), and pragmatic language (Capps et al., 1998; Volden, 1997). From the perspective of the ‘neurotypical’ perceiver, the individual with AS can be difficult to read, appearing idiosyncratic (Brewer et al., 2016; Froese et al., 2013) and disconnected from socio-cultural norms (Paul et al., 2009; Woodbury-Smith and Volkmar, 2009).

Social reasons emphasise how misunderstandings may originate through intersubjective processes (Kremer-Sadlik, 2004; Linell, 2009; Schegloff, 1992). Divergences of information and limited experiential overlap can make perspective-taking difficult (Gillespie and Martin, 2014; Jones and Nisbett, 1972). In order to avoid misunderstandings, both parties in a given relationship must work together to continually display their own understanding and probe the understanding of the other (Ichheiser, 1943; Schegloff, 1992). Thus, it is not only people with AS who need to explore the perspectives of FMs but also FMs who need to explore the perspectives of the person with AS. This need is furthered when one considers reports from people with autism who are able to articulate their perspectives, revealing how strongly they feel they are misunderstood because others do not know what it is like to be autistic (McGeer, 2004). The gap in mutual understanding is two-sided; however, there is a danger that FMs may not see the validity of such claims from PwAS because of their diagnosis, which, in turn, might exacerbate such misunderstandings. FMs, who scaffold perspective-taking in daily discourse for PwAS (Kremer-Sadlik, 2004), therefore play an important role in creating and addressing misunderstandings in PwAS–FM relationships.

Cultural reasons for misunderstandings highlight how Self–Other awareness is framed by normative expectations on what others ought to do given the circumstances (McGeer, 2001). What is distinct about PwAS–FM relationships is that both parties are aware of the diagnosis of AS, and the social construction of what that diagnosis means (i.e. the ways in which it is represented in culture) can ‘loop’ back into the very phenomena it seeks to describe (Hacking, 1996, 1999). Representations provide pre-packaged images and ideas about groups that are used by people to create default expectations about the behaviour and thinking of others (Schutz, 1932), and are significant for research on AS because of the divergent accounts of autism provided by science, the media and people with autism themselves (Kenny et al., 2016; Pellicano et al., 2014; Sarrett, 2011). For example, people with AS feel misrepresented by negative discourses associated with autism and disability (Bagatell, 2007), shaping how people with AS view themselves and others in relation to themselves (Parsloe, 2015). Such representations also impact those with whom they are intimately connected; for example, the ‘refrigerator parent’ theory of autism in the 1950s led to increased stigmatisation and guilt experienced by parents (Evans, 2013; Sousa, 2011). The looping effect therefore has a significant impact on self-identity in such relationships, as knowledge about the classification of autism changes the way those who are classified behave (Hacking, 1999, 2009; Sarrett, 2011) and leads neurotypical individuals to regulate behaviour in accordance with perceived norms (McGeer, 2009).

Representations can also affect how others are perceived. Research has shown that perspective-taking is ‘egocentrically anchored’, in the sense that perspective-taking begins with the assumption that Other has the same perspective as Self. Perspective-taking proceeds through serially adjusting from one’s own perspective, and such adjustments terminate when a plausible estimate is reached (Epley et al., 2004), reducing the ability to correct for additional biases in one’s immediate experience of others (Nickerson, 1998). Misunderstandings may therefore persist and remain unaddressed, because individuals seek explanations that conform to their own expectations, and will cease to probe beyond such explanations to discover its limitations.

To study empirically how representations of AS are differentially used by both people with AS and their FMs, we need a method that can study both sides of the social relationship.

### How can misunderstandings in relationships be identified?

The IPM (Laing et al., 1966) was developed to explore disagreements, perceived misunderstandings and actual misunderstandings in close personal relationships. According to this approach, social relationships are conceptualised as comprising direct perspectives (what Self and Other think about X) and meta-perspectives (each party’s estimation of what the Other thinks about X). Comparing these perspectives can reveal three dimensions: disagreements (i.e. comparing what Self and Other think about a given topic/person, for example, two people holding differing views about their relationship), actual misunderstandings (i.e. comparing what Self thinks about X with what Other thinks Self thinks about X, for example, a difference between one person’s perception of another’s view on the relationship, and the Other’s actual view on the relationship) and perceived misunderstanding (i.e. comparing what Self thinks about X with what Self thinks Other thinks about X, for example, one person anticipating that the Other holds a view about the relationship which differs from their own view).

This research builds upon the IPM methodology and focusses on perceived misunderstanding and actual misunderstanding. The ability to perceive the different perspectives of others enables actual misunderstandings to be addressed. These constructs are relevant to PwAS–FM relations because research suggests that PwAS will be limited in their ability to perceive differences in perspective from their own view (Frith and De Vignemont, 2005), leading to high levels of actual misunderstanding (as potential discrepancies in perspective between Self and Other remain unaddressed).

The IPM is therefore used to ask: (*RQ1*) what misunderstanding occurs in PwAS–FM relationships? And (*RQ2*), what reasons do participants give for perceived misunderstandings? *RQ1* is addressed by comparing numerical ratings of participants’ rating of Self and their predicted rating by Other (perceived misunderstanding), and participants’ predicted rating of Self by Other, and the rating they actually receive by Other (actual misunderstanding). *RQ2* is addressed by examining the reasons that PwAS and their FMs gave for particular ratings.

## Materials and methods

### Participants

Twenty-two PwAS and their chosen FMs were recruited from a charity supporting PwAS (n = 44; 22 dyads). Our inclusion criteria for the category of PwAS were broad given the challenges associated with diagnosis (Kaland, 2011; Leekam et al., 2000). Our criteria included (1) diagnosis for AS confirmed via contemporaneous reports (e.g. clinical records), or participants currently on the diagnostic pathway for AS having been referred for assessment by a medical professional, and (2) perceptual reasoning and verbal comprehension intelligence quotient (IQ) within the normal range (i.e. 70+; see supplementary file, section A). Our sample included a gender bias towards males (19:3), consistent with current rates of diagnoses for autism spectrum conditions (Taylor et al., 2013).

The main inclusion criteria for FMs were that they were responsible for the informal care needs of PwAS and did not have a formal diagnosis of an autism spectrum condition themselves. All of our dyads, except one (adult-cousin), involved adolescent/adult–parent relationships. (See Table 1 for details of participants.)

**Table 1.** Participant details.

PwAS		
Diagnosis	AS	18
	AS pathway	4
Age (range)		21.09 (16–41)
Gender M:F		19:3
IQ (range)		102.05 (72–128)
Living status	Independent	1
	Cohabiting	17
	Supported housing	2
	Unknown	2
Employment status	Full-time	1
	Part-time	2
	Apprenticeship	2
	Student (university)	2
	College (school/sixth form)	6
	Unemployed	9
Relative of PwAS		
Relationship to PwAS	Parent	21
	Cousin	1
Age (range)		53.27 (25–65)
Gender M:F		2:20

AS: Asperger’s syndrome; PwAS: People with Asperger’s syndrome; IQ: intelligence quotient.

### Materials

#### Contemporaneous reports

Reports including clinical reports, school reports and oral reports from staff at the charity, members and their parents were used to identify potential participants for the study.

#### The IPM topics and rating mats

A topic list was iteratively refined through five pilots using a combination of theory-driven concepts from the ADI-R (Le Couteur et al., 2006) and literature on AS. Topics reflected attested difficulties in communication including ‘small talk’ (e.g. difficulties starting interaction), ‘body language’ (e.g. reading non-verbal cues) and ‘managing discussion’ (e.g. dialogue and turn-taking). Narrow interests and systemised routines led to topics on adaptability, including ‘handling criticism’, ‘adapting routines’ and ‘sympathising’. Difficulties with future orientation (Howlin et al., 2004; Terrett et al., 2013) led to topics of ‘consequences of actions’, ‘organisation’ and ‘five-year view’. Finally, research on people with other communicative disabilities highlighted disagreements in perceptions of independence (Gillespie et al., 2010), leading to topics of ‘handle everyday tasks’, ‘make decisions (on own)’ and ‘visiting new places (on own)’. A context guide provided common examples (e.g. ‘everyday activities’ included washing up, food shopping and catching the bus) to help participants situate the meaning of the IPM items.

To complete the rating, participants used a six-point Likert scale from 0 to 5 (see supplementary file, section B). Topics were rated using an adapted version of ‘Talking Mats’ (Murphy, 2000) where participants were presented with items to be placed on the scale (an A3 mat divided into six scoring columns) from ‘is not at all good/often/easy’ (0) to ‘is very good/often/easy’ (5). This format was deemed preferable given its success in assessing people with aphasia, learning difficulties, dementia and brain injury, and the tendency for participants to adjust ratings as they evaluate questions (Moore and Gillespie, 2014).

### Procedure

Dyads were briefed together about the nature of the study before standard procedures concerning informed consent and a demographics questionnaire were completed. Participants were studied individually, with the sessions audio-recorded. Twelve topics were presented in a random order and rated using three different mats: (1) Self (e.g. rating themselves), (2) Other (e.g. rating their partner) and (3) Meta (e.g. rating how they perceive their partner will rate them).

The researcher made explicit the rating procedure for participants, saying ‘how good do you think you are at handling criticism?’ (rating mat 1), ‘How good do you think your relative is at handling criticism?’ (rating mat 2) and ‘How do you think your relative rated you for handling criticism?’ (rating mat 3). At the end of each rating, mat participants were offered the chance to adjust any ratings and to discuss any reflections. Debrief procedures completed the session. Ethical approval was granted by the researcher’s university and the charity where the research was conducted. Results were not returned to participants due to the potential for causing interpersonal issues associated with the discovery of misunderstandings.

### Analysis

To identify the misunderstandings that occur in PwAS–FM relationships (RQ1), we used numerical ratings to compare perceived misunderstandings and actual misunderstandings. Since the data rated were ordinal with non-normal distributions, the non-parametric Wilcoxon matched-pairs signed-ranked test was used.

To identify the reasons participants gave for misunderstandings (RQ2), we used transcribed audio recordings of the IPM interview using NVivo 10 (PwAS = 21; FM = 20; 3 participants declined to be recorded). Analysis focussed specifically on the explanations participants provided for perceived misunderstanding. A systematic approach of iterative categorisation was used (Neale, 2016), involving (1) open coding of participant explanations, (2) inductive sorting of codes into categories based on links between codes and (3) moving iteratively between data and coding framework to refine definitions into consistent and discrete categories. The unit of analysis included any meaningful segment of an utterance. FMs provided explanations for their ratings more frequently than PwAS (see Table 2), and thus, analysis focussed on the instances where participants did provide explanations and used the IPM scores to understand the magnitude of misunderstanding.

**Table 2.** Number of participants who provided an explanation for their rating when meta-representing their partner.

Group (N)	No explanation	Explanation < 6 topics	Explanation ⩾ 6 topics
AS (21)	11 (52%)	7 (33%)	3 (14%)
FM (20)	5 (25%)	9 (45%)	6 (30%)

AS: Asperger’s syndrome; FM: family member.

## Results

### RQ1: What misunderstandings occur in PwAS–FM relationships?

Table 3 presents average ratings from participants across all IPM topics. Wilcoxon matched-pairs signed-ranked tests with two-tailed significance were used to test for levels of significant perceived misunderstanding and actual misunderstandings. Results show that PwAS and FM perceived significant misunderstanding (PwAS: Z = −5.770, p < 0.001; FM: Z = −3.448, p = 0. 001). The results also indicate that both PwAS and FM did not experience significant actual misunderstanding (PwAS: Z = −0.378, p = 0.706; FM: Z = −1.018, p = 0.309).

**Table 3.** Do participants experience significant perceived misunderstanding and actual misunderstanding?

Group (N)	Average scores for rating target			Do participants perceive significant misunderstanding?^a^		Do participants experience significant actual misunderstanding?^b^	
	Self	Other	Meta	Z	Sig.	Z	Sig.
PwAS (21)	2.75	3.87	2.30	−5.770	< 0.001	−0.378	0.706
FM (20)	4.06	2.29	3.80	−3.448	0.001	−1.018	0.309

PwAS: People with Asperger’s syndrome; FM: family member.

a Calculated by comparing difference between rating of Self and predicted rating of Self by Other.

b Calculated by comparing difference between predicted rating of Self by Other and actual rating by Other.

Table 4 reports Wilcoxon matched-pairs signed-ranked tests to examine in further detail the perceived misunderstanding and actual misunderstanding of participants according to IPM topics (see supplementary file, section C, for median scores). Table 4 shows significant perceived misunderstanding, with PwAS expecting lower scores for ‘handling criticism’ (Mdn. 2 vs 2), ‘adapting routines’ (Mdn. 1 vs 2), ‘managing discussions’ (Mdn. 2 vs 3), ‘handling everyday tasks’ (Mdn. 3 vs 3) and ‘making decisions’ (Mdn. 2 vs 3). This result shows that PwAS are able to predict ratings about themselves that disagree with their own self-ratings. Accordingly, PwAS actually misunderstand FM on only one topic, ‘adapt routines’ (Mdn. 1 vs 3).

**Table 4.** Perceived misunderstanding and actual misunderstandings.

	Do PwAS perceive misunderstanding with FM about rating of PwAS?		Do FM perceive misunderstanding with PwAS about rating of FM?		Do PwAS misunderstand what FM thinks of PwAS?		Do FM misunderstand what PwAS thinks of FM?	
	Z	Sig.	Z	Sig.	Z	Sig.	Z	Sig.
Handle criticism	−2.266	0.023*	−1.687	0.092	−1.058	0.29	−2.854	0.004*
Adapt routines	−2.294	0.022*	−1.781	0.075	−2.459	0.014*	−0.992	0.321
Sympathy	−1.252	0.21	−2.215	0.027*	−0.884	0.377	−1.308	0.191
Small talk	−0.855	0.392	−2.047	0.041*	−0.912	0.362	−1.267	0.205
Body language	−1.299	0.194	−3.337	0.001*	−1.661	0.097	−1.356	0.175
Manage discussion	−2.623	0.009*	−1.895	0.058	−0.079	0.937	−1.175	0.24
Handle everyday tasks	−2.230	0.026*	−1.000	0.317	−0.022	0.982	−1.730	0.084
Make decisions (on own)	−2.430	0.015*	−0.758	0.448	−1.001	0.317	−0.263	0.793
Visit new places	−1.107	0.268	−0.265	0.791	−1.132	0.257	−1.713	0.087
Consequences of actions	−1.604	0.109	−2.273	0.023*	−1.767	0.077	−2.048	0.041*
Organisation	−1.363	0.173	−2.179	0.029*	−1.059	0.289	−0.254	0.799
Five-year view	−0.486	0.627	−0.032	0.974	−1.221	0.222	−0.497	0.619

PwAS: People with Asperger’s syndrome; FM: family member.

Asterisk (*) indicates statistically significant disagreement (p < 0.05).

Significant perceived misunderstanding across five topics is also shown by FM, although both high and low ratings were recorded. For ‘sympathy’ (Mdn. 4 vs 4), ‘body language’ (Mdn. 4 vs 5) and ‘consequences of actions’ (Mdn. 3 vs 4), FM perceived that they would be rated lower by PwAS than they had rated themselves. However, for ‘small talk’ (Mdn. 4 vs 4) and ‘organisation’ (Mdn. 4 vs 4), FM perceived that they would be rated higher by PwAS than they had rated for themselves.

Overall, these data show that misunderstandings occur on both sides of the relationship, and that perceived misunderstanding is more widespread than actual misunderstanding. PwAS correctly anticipated that their FMs would rate them lower in many regards, despite disagreeing with such views (i.e. rating themselves higher). This provides evidence of sophisticated perspective-taking, which researchers and FMs often assume is significantly compromised in individuals with AS (Sofronoff et al., 2014; Turowetz, 2015). PwAS represent themselves from the viewpoint of FM more negatively, which aligns with reports from people with autism about being misunderstood by others (Cederlund et al., 2010; McGeer, 2004). FM also perceived misunderstanding and rated PwAS lower than PwAS rated themselves, which is consistent with parent rating behaviour in other studies (Cederlund et al., 2010; Koning and Magill-Evans, 2001).

### RQ2: What reasons do participants give for perceived misunderstanding?

Systematic coding of transcripts revealed perceived misunderstanding due to reasons associated with Other (two subcategories) and Self. Table 5 provides definitions of the categories with accompanying examples, and Table 6 shows the coverage of categories across the participant sample.

**Table 5.** Categorisation of reasons provided by participants for perceived misunderstanding in the IPM.

Category	Subcategory	Definition	Illustrative Excerpts
The belief that the Other causes misunderstandings	Partial impairment in perspective-taking	Explanations which focus on narrow/restricted social understanding and perception.	FM7: He’s quite confident talking to new people. But then it does go to him talking at people. because that’s Asperger’s that’s what they are like. FM13: […] If I’m crying then he knows he has upset me but he doesn’t feel the connection. AS18 […] but she’ll just think ‘well you’re doing it in a different way than I would do it, so you’re doing it wrong, and I’ve got to sort that out’. AS14: I guess I think she sees me as being more attached to comfort zones than I necessarily am.
	Extreme impairment in perspective-taking	Explanations which focus on a complete barrier in introspection or perspective-taking with others.	FM12: He is totally dominated by himself really. FM8: I don’t think it will enter his head that I particularly think about the future. FM20: […] if he is having one of his meltdowns he doesn’t even think about the consequences of his actions. It’s just the here and now for that. FM19: I don’t think he has any idea what body language is so it probably doesn’t mean anything. AS: *No Cases*
The belief that the Self causes misunderstandings		Explanations where participants claim it is hard to read or imagine Others’ thoughts, or that the Self obscures being easily read by Others.	FM21: […] there are some scenarios where I don’t understand why she gets in a flap about things. FM2: Well, I don’t think he thinks I get him. And I possibly don’t get him. But I’m trying to. AS4: It’s hard to think. She doesn’t [.] it’s very difficult rating her. AS9: I’m trying to remember back over times when she does. I would say she does but sometimes I am just unreadable to her apparently.

IPM: Interpersonal Perception Method.

**Table 6.** Perceived causes of misunderstanding.

	PwAS (n = 21)		FM (n = 20)	
	No. of references	Percentage of participants	No. of references	Percentage of participants
1. Other a cause of misunderstanding	23	48	90	95
*1.1 Partial impairment in perspective-taking*	*23*	*48*	*65*	*95*
*1.2 Extreme impairment in perspective-taking*	*0*	*0*	*25*	*75*
2. Self a cause of misunderstanding	20	62	14	40

The diagnosis of AS was rarely mentioned by participants, perhaps to protect the positive identity of those diagnosed, or perhaps because it was so central to the relationships studied and the purpose for taking part in research it was deemed superfluous. However, representations do not need to be explicitly named in order to be used; rather, their use is evident in their effects (Moscovici, 2007). In this case, the differences in perceiving misunderstanding because of Self and Other evident in Table 6 show the representation of Asperger’s in use.

Table 6 highlights two ways of representing the Other. The first, ‘partial impairment in perspective-taking’, is two-sided as both PwAS and FM use this representation. The tendency to view others as biased in their social inferences is a common feature of interpersonal perception (Kruger and Gilovich, 1999; Pronin et al., 2002). In the PwAS–FM relationships studied, it was more frequently used by FM (95%) than PwAS (48%) which reflects the view of people with autism being impaired in the intersubjective understanding of others (Smukler, 2005; Solomon, 2015). However, it should be noted that PwAS perceived that they would not be fully understood by FM, which compares with reports from PwAS who feel their condition masks their true feelings towards FMs (Carrington and Graham, 2001).

Cases where participants perceived the Other to have an ‘extreme impairment in perspective-taking’ (i.e. claims that the Other is unable to introspect or imagine other minds) were one-sided as they were only used by FM to describe PwAS. This reveals how the representation of Asperger’s, in use by FM, licensed a more extreme dismissing of the perspective-taking abilities of the participants with AS. However, the data show that although participants with AS were less likely to provide reasons for perceived misunderstandings, most were able to reflect on Self and Other in the IPM.

Example 1 illustrates how the tendency for FM to use an ‘extreme impairment’ explanation, coupled with more nuanced social awareness of PwAS, sets up an actual misunderstanding:

*Example 1: Rating ‘consequences of actions’.*

*FM9, estimating Other’s rating. (FM9 predicts AS9 will rate her = 1; AS9 rates FM9 = 4)*.

103 BH: [How often] does he think you think about the consequences of your actions?

104 FM9: To be honest I don’t think he has thought about his own consequences.

105  I’ll go for one on that because I don’t think he will have ever thought about it to106  be honest.

107 BH: So he won’t=

108 FM9: =He won’t think about me he only thinks about himself really.

FM9 answers the IPM question (line 103) by representing AS9 as someone who experiences a complete absence of meta-representation (line 104/108). FM9 expects to receive a low rating from AS9 (= 1), because his difficulty in appropriately perceiving Self will result in an absent ability to perceive Other (lines 104–106). Yet FM9 overestimates the extent of impairment of AS9, given the rating actually provided by AS9 (=4) and his subsequent explanation:

*Example 2: Rating ‘consequences of actions’*.

*AS9, rating Self*.

9 BH: Ok. How often do you think about the consequences of your actions?

10 AS9: I think in the middle of the road. Sometimes I do things without thinking

11  and a lot of times there has been trouble. [participant rates 3]

*AS9, rating FM9*.

96 BH: How often does she [FM9] think about the consequences of her actions?

97 AS9: I think she knows that she is really good at thinking what will happen if

98  something is taken out of context. She makes sure people understand her in

99  the way that she wants to be understood. If there was something wrong she

100  probably wouldn’t say it out [a]loud to their face. [participant rates 4]

Although AS9 confirms FM9’s expectation that ‘consequences of actions’ are a source of difficulty for him (line 11), it is not to the extent that it prevents him from appreciating the same skill in other people. AS9 articulates a clear difference between experiencing the challenges of ‘consequences of actions’ and observing ‘consequences of actions’ in FM9. He is not anchored egocentrically in the way FM9 predicts but rather shows a much more nuanced and detailed understanding of how ‘consequences of actions’ apply differently to Self and Other, coupled with an awareness of how FM9’s thoughts and intentions are shaped by different situations (line 97–100).

Examples 1 and 2 therefore highlight an actual misunderstanding where FM9 overestimates the social impairments of AS9. The origins of overestimation may be both interpersonal and cultural. Parents of children with AS report levels of elevated stress (Epstein et al., 2008) and are more likely to underestimate the social skills of adolescents with autism compared with neurotypical parents (Kuo et al., 2011). Frustration with past misunderstandings may explain why FM over-estimate the extent of impairment in perspective-taking of PwAS. However, other FM who used an extreme view of impairment to describe PwAS connected it explicitly to the expectations the diagnosis of AS sets up:

*Example 3: Rating ‘Managing discussions’*.

*FM14, rating AS14 (FM14 predicts AS14 will rate her = 3; AS14 rates FM14 = 4)*.

83 FM14: […] Like the small talk thing he probably wouldn’t notice because

84  he’s not that good at it himself so he wouldn’t see that in somebody else.

85  Whereas I know I am actually not that good.

86 BH: So you’re anticipating a sort of misunder-standing about that in a way?

87 FM14 Yea because of his autism [.] yea.

Example 3 highlights how FM14 perceives misunderstanding based on normative expectations about an autism diagnosis involving an inability to understand others (line 87). Such expectations may adversely affect interpersonal perspective-taking because it prevents FMs from seeking out further explanations for why misunderstandings occur, leading to the nuanced social ability of PwAS being overlooked. Although one FM claimed ‘the more I learn about Asperger’s the more I can understand AS17’ which she reported led to increased patience and the ability to ‘respond differently’, Example 3 shows that the diagnosis can also have negative effects, reinforcing low expectations about PwAS.

FM under-estimating PwAS was acknowledged by FM4 as a problem, ‘He came to me the other day and said “You’ve been doing really well mum”, and I thought “Oh God”. So sometimes there is a lot more going on than you think there is’. In the absence of normative social feedback, FM may have to resort to using culturally based assumptions about PwAS to interpret their behaviour. This perhaps explains why so many FMs used representations of perspective-taking impairment to describe PwAS (see Table 5). Perceptions of extreme perspective-taking impairment may be a simplification of the cognitive view on autism (McGeer, 2004; Sarrett, 2011), which theorises that perspective-taking difficulties originate from a defective capacity to represent other minds (Boucher, 2012). Here, we are not dealing with whether some approaches to autism and Asperger’s over-emphasise immutable cognitive variables (see McGeer, 2004; Sarrett, 2011), but rather with the way in which these cognitive theories become popular representations, used by PwAS and their FM. These representations loop back into perspective-taking by potentially cutting short peoples’ efforts to serially adjust from their own perspective to more adequately approximate the perspectives of PwAS.

Overall, the explanations for misunderstanding provided by participants showed a strong tendency to focus on the limitations of PwAS, with many FMs perceiving an extreme impairment in social understanding. While this is congruent with the characterisation of people with autism as having a lack of self-awareness and a complete inability to understand others (Sarrett, 2011), such beliefs prevent FM from considering the more nuanced aspects of PwAS behaviour. Evidence for this is shown by some of the detailed explanations provided by PwAS, which demonstrate the capability to imagine the subjectivity of others across different contexts, despite FM broadly claiming that this would not be possible. PwAS also showed a greater propensity to reflect on Self as the possible cause of misunderstanding much more than FM (62% vs 40%). Thus, although, PwAS provided less detailed and less frequent explanations of misunderstandings, comparing with findings from other interview data (Capps et al., 1998), they are not as limited as FM assume in their ratings and explanations.

## General discussion

We used an adapted version of the IPM (Laing et al., 1966) designed for exploring interpersonal relations, to examine two-sided misunderstandings in PwAS–FM relations. This research makes empirical and methodological contributions.

The quantitative finding showed that PwAS correctly anticipated that their FMs would rate them lower in many regards, despite the fact that they disagree with their view (i.e. rating themselves higher). This was further supported by the qualitative finding where, in some cases, PwAS were able to imagine that FM would rate PwAS poorly, often overgeneralising the extent of their social limitations. The finding extends research showing that PwAS are able to recognise and see the problems inherent in their own diagnosis of AS (Cederlund et al., 2010) by highlighting the unrealised potential for PwAS to also take the perspective of others.

The finding also furthers the discussion about theory of mind in people with Asperger’s (Peterson et al., 2009), because PwAS were able to accurately predict FM ratings but showed a much lower tendency to articulate the reasons for such scores to the researcher (i.e. there is perspective-taking but less perspective-sharing). Clearly, PwAS have theories about the minds of their FMs, but articulating this to a researcher outside of the family context presents more of a challenge. This highlights why there may be a validity gap between laboratory and naturalistic assessments of the ability to take perspective (Verhoeff, 2015), namely, because theory of mind experiments focus on imputing mental states (i.e. perspective-taking) not on communicating and displaying one’s own perspective (i.e. perspective-sharing).

The IPM also shows that making oneself readable to others may not be straightforward for PwAS. Perceptions of extreme perspective-taking impairment used by FM act as a confirmatory bias (Nickerson, 1998), preventing FM from probing further about the causes of interpersonal misunderstanding as evidence is interpreted in a way that is partial to their existing beliefs. Thus, these representations loop back (Hacking, 2009) into the phenomena they purport to describe, potentially leading to the more nuanced behaviour of PwAS being overlooked.

The two-sided nature of misunderstandings evident in PwAS–FM relationships highlights the importance of how we design concepts and apply them to people. Parent interviews and questionnaires play an integral role in evaluating whether or not their children have sufficient problems to warrant a diagnosis of AS, and previous studies have shown a disparity between the perspectives of PwAS and parents (Cederlund et al., 2010). Our data highlight the need to place such perspectives on equal footing because misunderstandings can be two-sided, and reports from people with AS have social validity, despite their diagnosis. In addition, diagnoses extend beyond improving access to support and services (Kite et al., 2013), to impact identity in both positive (Chell, 2006; Parsloe, 2015) and negative ways (Broderick and Ne’eman, 2008; Sarrett, 2011; Smukler, 2005) within social relationships (Powell and Acker, 2016). Our study provides evidence about how the representation of AS loops back to operate at the interpersonal level, shaping how relations are perceived and managed by framing normative expectations about perspective-taking, specifically for FM. Knowledge about diagnoses can act as a turning point in the parents’ journey of understanding their children (Robinson et al., 2015). It therefore follows that the findings of this article, specifically that FMs over-estimate the impairment of PwAS and that this is supported by their representations of AS, can itself be relayed back to parents at the point of diagnosis to mitigate the impact of confirmatory biases.

In addition to empirical contributions, the two-sided IPM methodology contributes to the methodological toolkit for understanding PwAS for three reasons. First, it situates social understanding within significant and familiar social relations, enabling researchers to overcome the validity gap between abstract assessments and real-world phenomena (Verhoeff, 2015) and to study the production and circulation of knowledge about autism in family settings (Solomon, 2010). Second, it places PwAS and FM on an equal footing, avoiding the risk of reinforcing expectation about misunderstandings originating from PwAS (Turowetz, 2015). Finally, this two-sided methodology is a form of targeted ethnography that allows the origins of misunderstandings to be identified and to be potentially used to develop interventions.

## Limitations

Most relationships involved mother–son relationships, and thus the findings may not account for gender differences when parenting children with autism (Jones et al., 2013), such as daughters who are sometimes perceived to have greater impairments as a result of higher parental expectations (Holtmann et al., 2007; McLennan et al., 1993). Likewise, girls with AS have a different behavioural phenotype to boys (Rivet and Matson, 2011) with greater language and social skills (Kopp and Gillberg, 1992) which can mask their condition and impact the ratings provided in the IPM. Participants were also recruited via a charity, and thus, the sample reflects the population of PwAS who were willing to take part in research and also had FMs who were accessible. Not captured within the data are PwAS–FM relationships where misunderstandings have become so severe that the relationships have broken down. Also, FM could possess traits of AS (Nydén et al., 2011) which would potentially impact their ability to perceive misunderstanding. Resource constraints prevented assessments of FM, although any potential traits were not significant enough to prevent parents from living independent lives and finding employment.

The study assessed topics related to social skill; however, interpersonal relations involve perspective-taking about other phenomena, such as likes/dislikes and political views. PwAS also exhibit better social skills when social cues are made explicit (Senju et al., 2009), and thus, the semi-structured interview style of the IPM may not capture the full complexity of everyday misunderstandings that PwAS experience. Since misunderstandings are interactionally achieved, rather than purely cognitive mistakes, analysing real-life interactions between PwAS and FM would yield important further insights about how misunderstandings are negotiated and integrated into shared understanding.

The two-sided methodology used here has potential to be used, and adapted, alongside existing measures of AS in order to develop a more holistic understanding of (1) the origins of misunderstanding in relationships involving PwAS and (2) how an AS diagnosis impacts misunderstandings within these social relations.

## Conclusion

The empirical contribution of the study has shown that people with Asperger’s are able to predict lower scores from FMs, despite disagreeing with their view, and that FMs often over-estimate the extent to which relatives with AS are egocentrically anchored in their own perspective. The methodological contribution of the study demonstrates that a two-sided methodology is viable and can be used to identify social processes that both support and hinder social understanding within relationships affected by a diagnosis of AS.

## Supplementary Material

Supplementary Material
