# Supplementary material for: Perspective-taking is two-sided: Misunderstandings between people with Asperger’s syndrome and their family members
Source: Autism. 2017 Jul 7;22(6):740–50. doi: 10.1177/1362361317708287 (PMC6055325; doi:10.1177/1362361317708287)
Supplement: Supplementary Material [file AUT708287_Supplementary_Material.pdf]

Supplementary material

Section A

Wechsler Scale of Intelligence (WASI-II). The Wechsler Abbreviated Scale of Intelligence, Second Edition (WASI-II) was administered to participants with Asperger's syndrome to ensure there was no underlying learning difficulty. The Wechsler scale uses four sub-tests (Vocabulary, Similarities, Block Design, and Matrix Reasoning) to provide a brief and reliable measure of cognitive ability.

Section B

Talking Mats Example

An example of rating mat 2 (FM rating PwAS). Before each mat the researcher explained the perspective being captured (e.g. Self, Other or Meta) and adjusted the questions accordingly (e.g. “how would you rate yourself on topic X?”, “how would you rate your partner on topic X?”, “how do you think your partner will rate you on topics X?”).

Materials were laminated to make it easy to slide the topics around. Topics were also colour coded into groups of three: Blue = adaptability; Green = communication; purple = independence; yellow = future orientation. Colour coding the topics made the task more visually interesting for participants, and made it easy for the researcher to spot missing topics. Finally, all topics were numbered in order to assist the researcher in documenting the results in a systematic way.

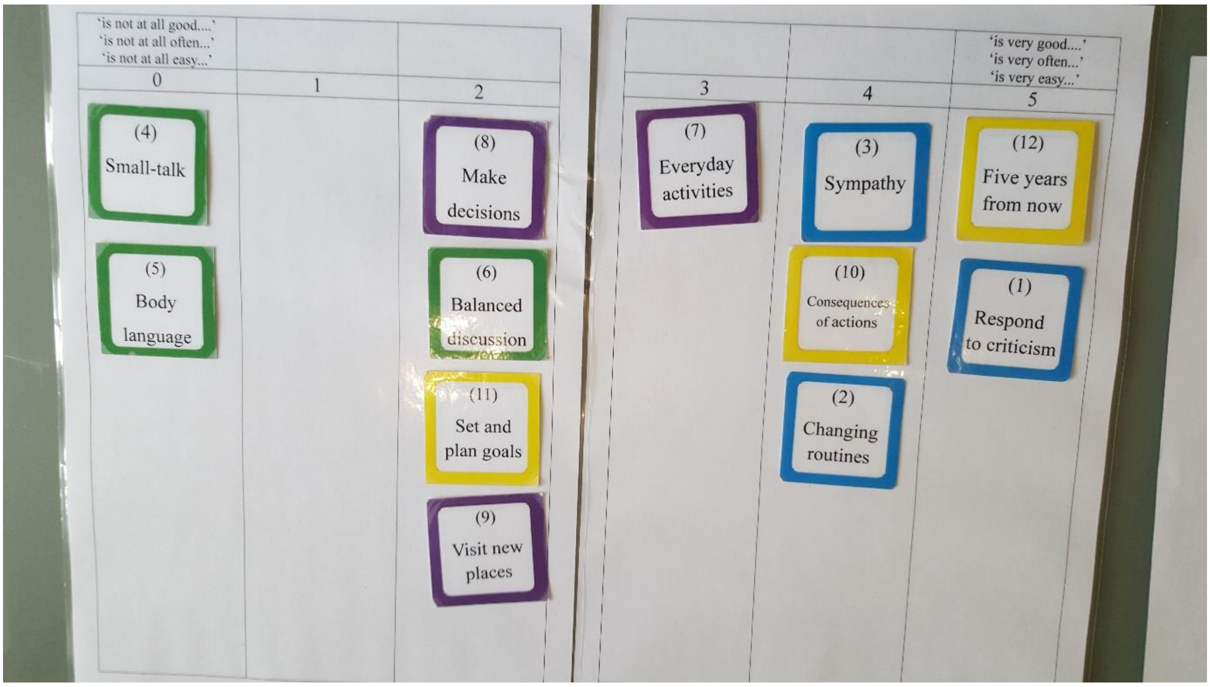

Section C

IPM ratings produced by people with Asperger's (PwAS) and family members (FM)

	Rating Self (e.g. "How good are you at X?")		Rating Other (e.g. "How good is your partner at X?")		Rating Meta* (e.g. How do you think your partner will rate you for X?"	
	Median (range)		Median (range)		Median (range)	
	PwAS	FM	PwAS	FM	PwAS	FM
**Handling criticism**	2 (0-5)	3 (0-5)	3 (1-5)	1 (0-4)	2 (0-4)	2 (0-4)
**Adapting routines**	2 (0-4)	5 (3-5)	4 (1-5)	3 (0-5)	1 (0-3)	4 (1-5)
**Sympathy**	3 (1-5)	4 (3-5)	4 (2-5)	2 (1-4)	3 (0-5)	4 (1-5)
**Small talk**	3 (0-5)	4 (1-5)	4 (1-5)	2 (0-5)	3 (0-5)	4 (2-5)
**Body language**	3 (0-5)	5 (2-5)	4 (2-5)	2 (0-4)	2 (0-4)	4 (1-5)
**Managing discussions**	3 (0-5)	4 (2-5)	4 (1-5)	2 (0-5)	2 (0-4)	4 (1-5)
**Handling everyday tasks**	3 (1-5)	5 (4-5)	5 (3-5)	3 (0-5)	3 (1-5)	5 (4-5)
**Making own decisions**	3 (0-5)	5 (3-5)	4 (2-5)	2 (0-5)	2 (0-5)	4 (3-5)
**Organisation**	3 (0-5)	4 (2-5)	4 (0-5)	2 (0-5)	3 (0-5)	4 (3-5)
**Visit new places**	3 (1-5)	5 (2-5)	4 (3-5)	3 (0-5)	3 (0-5)	5 (3-5)
**Consequences of actions**	3 (0-5)	4 (2-5)	4 (2-5)	2 (0-5)	2 (0-5)	3 (1-5)
**Five year view**	3 (0-5)	4 (1-5)	4 (1-5)	3 (0-5)	2 (0-5)	3 (1-5)

* Meta = what person A thinks person B thinks of person A
